# Risk factors associated with adverse metabolic health in youth with perinatally acquired HIV living in the United Kingdom

**DOI:** 10.1097/QAD.0000000000004533

**Published:** 2026-06-02

**Authors:** Caroline Foster, Alexandra Blenkinsop, Merle Henderson, Hermione Lyall, Sarah Fidler

**Affiliations:** a 900 Clinic; b Department of Paediatric Infectious Diseases, Imperial College Healthcare NHS Trust; c Department of Mathematics, Imperial College London; d Department of Infectious Diseases, Imperial College London, Imperial College NIHR BRC, London, UK.

**Keywords:** antiretroviral therapy, metabolic dysfunction-associated steatotic liver disease, metabolic syndrome, perinatally acquired HIV, youth

## Abstract

**Objective:**

Non-AIDS-related morbidity and mortality in people with HIV are associated with adverse metabolic health, influenced by both traditional-related and HIV-related risk factors. There is a paucity of data for youth with perinatal HIV (PHIV). We explored the relationship between markers of metabolic health and antiretroviral therapy (ART) in youth with PHIV.

**Design:**

Longitudinal observational cohort study; 26 months.

**Methods:**

Eighty-five youth enrolled in the ‘Bone Density in Youth living with PHIV’ (BONDY) underwent assessment of metabolic health including fasting biochemistry, BMI, total body dual energy x-ray absorptiometry scan and hepatic transient elastography.

**Results:**

Of 85 participants with PHIV, mean age 21.7 years, 58% were female and 82% black African. Median ART exposure was 15 years, median CD4^+^ cell count 623 cells/μl at enrolment with 82% viral load less than 200 copies/ml. Median weight gain over 26 months was 3 kg, with BMI category overweight/obese increasing from 37 to 50% with 48% having dyslipidaemia. Metabolic syndrome criteria were fulfilled in 7%, with 13% having hypertension, 18% hepatic steatosis, and 7% fibrosis on transient elastography. Bayesian regression analyses demonstrated no association of integrase strand transfer inhibitor (INSTI) and/or tenofovir alafenamide (TAF) use, and sex at birth or prior CDC-C diagnoses or current HIV viraemia on metabolic outcomes.

**Conclusion:**

While adverse metabolic outcomes are common in this youth cohort with PHIV, no association was observed with INSTI and/or TAF use. Given the relatively young age of this cohort, preventative interventions targeting traditional metabolic risk factors are required to avoid comorbidities in later life.

## Introduction

Metabolic syndrome (MetS), characterized by central obesity, hypertension and impaired lipid and glucose metabolism, is a predictor of future cardiovascular risk and type 2 diabetes (T2DM). Increased rates of MetS are reported in adults with horizontally acquired HIV (HHIV); associated with traditional factors including obesity, alcohol, smoking, sedentary lifestyle and genetic factors – and HIV-related risk factors including viraemia and some antiretroviral regimens [1]. People born with perinatally acquired HIV (PHIV) experience lifelong exposure to HIV-associated inflammation and increasingly antiretroviral therapy (ART), with the oldest entering their fifth decade of life; approaching the age clinical cardiovascular events and MetS-associated T2DM appear in the general population [2–5].

Emerging data suggests increased rates of MetS and cardiovascular (CVS) risk in PHIV. Cohorts from the United States report incidence rates of T2DM of 19%, dyslipidaemia 50% and hypertension 22% by 30 years of age [6]. In this setting, youth with PHIV (median age 26 years) had higher rates of MetS (13 vs. 4%), than aged/ethnically matched HIV-negative youth, and higher rates of truncal/android fat distribution; a pattern associated with increased CVS events [7]. While two-thirds were overweight/obese, rates were comparable to youth without HIV (47 vs. 51% overweight, 20 vs. 17% obese). In settings with lower rates of obesity, youth with PHIV remain at increased risk of MetS (France; 13.2% males, 10.4% females with PHIV vs. 10.6% and 1.7% youth without HIV and in 10.2% Thai PHIV youth) [8,9]. Conversely data from South Africa in youth, median age 18 years, suggested lower rates of MetS in those with PHIV (12%) when compared to youth with HHIV (34%) and HIV negative youth (23%) but with higher rates of dyslipidaemia in youth with PHIV without obesity and highlights the considerable geographic heterogeneity in obesity and metabolic health [10].

Obesity-driven metabolic dysfunction-associated steatotic liver disease (MASLD) is the main driver of liver fibrosis in adults with HHIV [11]. Transient elastography (FibroScan)-controlled attenuation parameter (CAP) scores have good correlation with steatosis in adults with HHIV and in youth without HIV [12,13]. Early data in adolescents with PHIV suggests increased rates of steatosis compared to HIV-negative youth, only partially explained by metabolic factors [14,15].

The potential metabolic impact of ART includes dyslipidaemia with boosted protease inhibitors (bPI), and weight gain with integrase inhibitors (INSTI) and tenofovir alafenamide (TAF) [4,16–18]. Data for PHIV is emerging; 70% of Thai adolescents receiving bPIs had dyslipidaemia, with tenofovir disoproxil fumarate (TDF) a protective factor [19]. The bPI use remains high for treatment-experienced youth with extensive drug resistance [20,21]. Studies of INSTI-related weight change in PHIV vary from no association to weight gain in young women with increased truncal fat deposition [4,22–25]. In a meta-analysis of four paediatric TAF studies, weight-for-age *z* scores increased by 0.25 at 48 weeks; however, data on the impact of INSTIs with TAF in youth is lacking [26].

The Reprieve Study demonstrated a 35% reduction in major CVS events following statin use in adults with HHIV (median 50 years) and a low/moderate CVS risk, acknowledging that cardiovascular risk scores underestimate risk in those with HIV and in younger populations [27,28]. Interventions to reduce risk factors for non-communicable diseases are required from early adult life, prior to established pathology [29]. In this study, we examined the relationship between markers of metabolic health, hepatic steatosis, HIV clinical status and ART exposure in PHIV youth in the United Kingdom, exploring the hypothesis that weight gain associated with INSTIs in conjunction with TAF would impact metabolic health.

## Methods

### Study population

The ‘Bone Density in Youth living with perinatally acquired HIV’ (BONDY) study has been described in full previously [30]. Participants aged 15–24 years were eligible for the longitudinal study arm with a second assessment after 24 months. At this visit, they were invited to enrol in the sub-study (BONDY-Met) assessing metabolic health.

### Study procedures BONDY-Met

Demographics, ART history, past CDC-C diagnoses, length of viral suppression, latest CD4^+^ cell count, HIV viral load, a family history of diabetes, cardiovascular and liver disease, concomitant medication and substance use were recorded [30]. During the study period, the NHS commissioned TAF for those younger than 25 years, with a TDF to TAF switch offered to participants and all ART changes documented [31].

Height, weight, Tanner staging, blood pressure (BP) and waist circumference were assessed. BMI was calculated as weight/height (kg/m^2^) and as an age-adjusted *z* score if 15-19 years and classified as overweight BMI greater than than 25–29 or obese BMI greater than 30.

#### Metabolic biochemistry

Participants fasted for 12 hours, and glucose, lipid profile, glycosylated haemoglobin (HbA1c), C-peptide, insulin and liver enzymes measured. Homeostatic model for assessment of insulin resistance (HOMA-IR) was calculated using insulin (μU/ml) × glucose (mmol/l)/22.5 [32].

Total body dual energy X-ray absorptiometry (DXA) scans measured total body fat, android (AFM) and gynoid fat mass (GFM), using Lunar Prodigy or iDXA (GE Healthcare) scanners [30]. The android : gynoid ratio (AGR) was calculated using the fat percentage in regions A and G.

### Transient elastography (FibroScan)

Assessed CAP (dB/m) with correlates of steatosis graded as [33].

1. 238–260 dB/m S1 = 11 33% liver fatty change (LFC)
2. 260–290 dB/m S2 = 34–66% LFC
3. >290 dB/m S3 = 67+% LFC

Fibrosis scoring (F) assessed by E score (kPa) and graded as:

1. E2-7 – F0-1 no/mild fibrosis
2. E7-10 – F2 moderate
3. E10-14 – F3 severe
4. E14+ – F4 cirrhosis

### Metabolic syndrome

Rates were defined using a consensus definition adjusted for age and ethnicity [34]. Three of five criteria led to a diagnosis of MetS.

1. Waist circumference >102 cm men, >88 cm women (16+ years)
2. Triglycerides ≥1.7 mmol/l
3. HDL <1.04 mmol/l men, <1.29 mmol/l women
4. Blood Pressure ≥130/85 mmHg
5. Fasting glucose ≥5.6 mmol/l

### Statistical analysis

Participant characteristics were summarized using counts and proportions for categorical variables, means and standard deviations (SD) for normally distributed continuous variables, and medians with interquartile ranges (IQR) for skewed distributions. We examined six primary outcomes: change in BMI baseline to follow-up, and five metabolic outcomes at visit 2 (FibroScan CAP, HbA1c, HOMA-IR, MetS, and meeting two criteria for MetS). Associations of INSTI-based vs. other ART, TAF-containing vs. non-TAF-containing regimens, INSTI + TAF vs. other regimens and HIV parameters (CDC-C, HIV viraemia and CD4^+^ : CD8^+^ ratio) were estimated using Bayesian generalized linear models with Gaussian link functions for continuous outcomes and logit link functions for binary outcomes. All models were adjusted for age and sex, and models for BMI change also adjusted for baseline BMI. For each outcome, we quantified the evidence for an association by estimating the posterior probability that the corresponding regression coefficient exceeded 0 for continuous outcomes or 1 (on the odds ratio scale) for binary outcomes. The threshold for significance was set at 1% (one-sided) due to multiple hypothesis testing increasing the probability of a spurious finding, with probabilities greater than 99% interpreted as significant. Potential interactions between treatment and sex were assessed.

### Ethical considerations

All participants provided written consent with ethical approval from the London-Stanmore Research Ethics Committee (REC) (IRAS 18/LO/1474).

## Results

Of 100 youth eligible for the BONDY study longitudinal arm (perinatal HIV age 15–24 at study entry, on ART), 85 enrolled in BONDY-Met: 49 (58%) were female, 70 (82%) of black ethnicity with a mean age of 21.7 years (SD 2.7). Reasons for not enrolling included (*n* = 15): transfer of care 5, personal choice/did not attend 5, unable to consent: pregnant 1, psychosis 1, central nervous system infection 1, travel 1, incarceration 1). The median follow-up was 26 months (IQR 25, 28) from study entry with a median weight gain of 3 kg [IQR −1, 7]. At latest follow-up, median CD4^+^ cell count was 623 cells/μl (IQR 467, 770), with 68 (80%) having a viral load less than 200 copies/ml. Mean ART duration was 15 years with 39 (46%) currently on INSTI-based ART, 32 (38%) on bPI [darunavir 27, atazanavir 5 (1 unboosted)] with 43 (51%) on TAF and 14 (16%) on INSTI +TAF (Table 1). Half had a prior CDC-C diagnosis, 22% were current or former smokers, 18% had a family history of cardiovascular disease, 26% a family history of diabetes mellitus and 32% drank alcohol. All were postpubertal and none had diabetes mellitus or were receiving lipid-lowering or antihypertensive agents.

Median weight gain over 26 months was 3 kg, with BMI category overweight/obese increasing from 37 to 50% with 28 (33%) having an A/G ratio within the third tertile. Forty-one (48%) had MetS defined dyslipidaemia and 11 (13%) had hypertension [39]. Six (7%) fulfilled MetS criteria with a further 13/85 (15%) having two criteria. Insulin resistance, (HOMA-IR ≥3) was evident in 19% with none having an HbA1c above 48 mmol/l (Table 1). On transient elastography, 15 (18%) had evidence of steatosis (≥S2), 7 (8%) had evidence of fibrosis (≥F2), and 8% had liver transaminases above the upper limit of normal. Mean BMI was 24.7 (5.1) with no difference in change from baseline by TAF (*n* = 43) vs. non-TAF/TDF (*n* = 40).

**Table 1:** Participant characteristics.

Characteristic		Total (*n* = 85)	Female (*n* = 49)	Male (*n* = 36)
Age (years)	Mean (SD)	21.7 (2.7)	21.6 (2.8)	21.9 (2.7)
Ethnicity	Black/African/Caribbean/Black British	70 (82%)	39 (80%)	31 (86%)
	Other	15 (18%)	10 (20%)	5 (14%)
ART duration (years)	Mean (SD)	15.0 (6.1)	14.3 (6.8)	15.8 (5.2)
ART anchor	INSTI-based	39 (46%)	28 (57%)	11 (31%)
	PI-based	32 (38%)	14 (29%)	18 (50%)
	NNRTI-based	14 (16%)	7 (14%)	7 (19%)
TAF/TDF	TAF	43 (51%)	28 (57%)	15 (42%)
	TDF	2 (2%)	0 (0%)	2 (6%)
	Non-TAF/TDF	40 (47%)	21 (43%)	19 (53%)
CD4^+^	CD4^+^ (cells/μl) (median [IQR])	623 [467, 770]	650 [524, 791]	583 [436, 736]
CD4^+^: CD8^+^ ratio	Median [IQR]	0.9 [0.6–1.3]	0.9 [0.6–1.4]	0.9 [0.6–1.1]
Viral load (copies/ml)	<20	57 (67%)	30 (61%)	27 (75%)
	20–199	11 (13%)	8 (16%)	3 (8%)
	≥200	16 (19%)	10 (20%)	6 (17%)
Previous CDC-C	Yes	44 (52%)	26 (53%)	18 (50%)
Smoking history	Smoking never	66 (78%)	39 (80%)	27 (75%)
	Smoking current/former	19 (22%)	10 (20%)	9 (25%)
Any alcohol		27 (32%)	15 (31%)	12 (33%)
Family history of CVD	15 (18%)	9 (18%)	6 (17%)	
Family history of DM	22 (26%)	11 (22%)	11 (31%)	
Metabolic parameters
BMI (baseline)^a^	Underweight	1 (1%)	0 (0%)	1 (3%)
	Normal weight	53 (62%)	27 (55%)	26 (72%)
	Overweight	22 (26%)	17 (35%)	5 (14%)
	Obese	9 (11%)	5 (10%)	4 (11%)
BMI (follow-up)^a^	Underweight	2 (2%)	0 (0%)	2 (6%)
	Normal weight	40 (47%)	21 (43%)	19 (53%)
	Overweight	24 (28%)	16 (33%)	8 (22%)
	Obese	19 (22%)	12 (24%)	7 (19%)
Prevalence of metabolic syndrome	6 (7%)	5 (10%)	1 (3%)	
Prevalence of dyslipidaemia	41 (48%)	28 (57%)	13 (36%)	
Prevalence of hypertension	11 (13%)	5 (10%)	6 (17%)	
Waist circumference (cm)	Mean (SD)	80.6 (14.4)	80.4 (13.4)	80.8 (16.0)
SBP	Mean (SD)	120.3 (12.5)	116.5 (11.0)	125.6 (12.6)
DBP	Mean (SD)	75.8 (10.5)	75.5 (9.5)	76.1 (11.8)
Triglycerides	Mean (SD)	0.8 (0.5)	0.8 (0.4)	0.9 (0.6)
	≥1.7 mmol/l	6 (7%)	5 (10%)	1 (3%)
Total cholesterol	Mean (SD)	4.2 (0.9)	4.1 (0.9)	4.3 (1.0)
	≥5.18 mmol/l	14 (16%)	7 (14%)	7 (19%)
HDL-cholesterol	Mean (SD)	1.4 (0.4)	1.4 (0.4)	1.3 (0.4)
	<1.29 (M), <1.04 (F)	31 (36%)	24 (49%)	7 (19%)
LDL-cholesterol	Mean (SD)	2.6 (0.7)	2.5 (0.6)	2.7 (0.7)
	≥3.4 mmol/l	11 (13%)	5 (10%)	6 (17%)
Glucose	Mean (SD)	4.5 (0.5)	4.5 (0.4)	4.6 (0.5)
	>=5.6 mmol/l	1 (1%)	1 (2%)	0 (0%)
HOMA-IR	Mean (SD)	2.4 (2.0)	2.5 (2.2)	2.2 (1.6)
	≥3.0	16 (19%)	9 (19%)	7 (19%)
HbA1c	Mean (SD)	33.0 (4.5)	32.2 (4.1)	34.0 (4.8)
	>48 mmol/l	0 (0%)	0 (0%)	0 (0%)
C-peptide	Mean (SD)	589.6 (333.8)	588.7 (328.5)	590.8 (346.6)
	>1500 pmol/l	2 (3%)	1 (2%)	1 (3%)
Insulin	Mean (SD)	11.7 (8.7)	12.1 (9.4)	11.3 (7.6)
	>15 μU/ml	14 (18%)	7 (16%)	7 (22%)
ALT	Mean (SD)	25.6 (21.7)	18.5 (6.6)	35.3 (30.1)
	>45 (M), >34 (F)	7 (8%)	2 (4%)	5 (14%)
Total body dual energy X-ray absorptiometry
Total body fat (%)	Mean (SD)	33.9 (10.8)	39.0 (7.5)	26.5 (10.8)
Android (A) fat (%)	Mean (SD)	34.0 (14.4)	38.7 (11.9)	27.4 (15.1)
Gynoid (G) fat (%)	Mean (SD)	37.1 (12.6)	43.7 (7.7)	27.9 (12.4)
A/G ratio^a^	Mean (SD)	0.9 (0.2)	0.9 (0.2)	0.9 (0.2)
	Tertile 1	29 (34.5%)	18 (36.7%)	11 (31.4%)
	Tertile 2	27 (32.1%)	16 (32.7%)	11 (31.4%)
	Tertile 3	28 (33.3%)	15 (30.6%)	13 (37.1%)
Transient elastography (FibroScan)
CAP (dBM)	Mean (SD)	198.9 (52.9)	196.8 (49.9)	201.8 (57.4)
Steatosis	S1 (238–260)	4 (4.7%)	3 (6.1%)	1 (2.8%)
	S2 (260–290)	5 (5.9%)	3 (6.1%)	2 (5.6%)
	S3 (290–400)	6 (7.1%)	2 (4.1%)	4 (11.1%)
E-score (kPA)	Mean (SD)	5.3 (3.6)	4.9 (2.6)	5.9 (4.7)
	2–7	78 (91.8%)	45 (91.8%)	33 (91.7%)
	7–10	4 (4.7%)	3 (6.1%)	1 (2.8%)
	10–14	0 (0.0%)	0 (0.0%)	0 (0.0%)
	≥14	3 (3.5%)	1 (2.0%)	2 (5.6%)

ART, antiretroviral therapy; ALT, alanine aminotransferase; BP, blood pressure; BMI, body mass index^a^ z scores aged 15–19 years; CAP, controlled attenuation parameter; CDC-C, Centre for Disease Control category C AIDS diagnostic condition; CVD, cardiovascular disease; DM, diabetes mellitus; E-score, elastography score; F, female; HbA1c, glycosylated haemoglobin; HDL, high-density lipoprotein; INSTI, integrase strand transfer inhibitor; IQR, interquartile range; LDL, low-density lipoprotein; M, male; NNRTI, non-nucleoside reverse transcriptase inhibitor; PI, protease inhibitor; SD, standard deviation; TAF, tenofovir alafenamide; TDF, tenofovir disoproxil fumerate.

a Central fat deposition assessed by DEXA as an android (abdominal)/gynoid (hip) ratio of ≥0.8 in women and ≥1.0 in men has been associated with an increased risk of adverse metabolic and cardiovascular outcomes in youth and adult populations [34].

Bayesian regression analyses demonstrated no association of INSTI and/or TAF containing ART on metabolic outcomes including, BMI change, MetS, hepatic steatosis, HbA1c and HOMA-IR (Table 2). Further analyses showed no evidence of interactions between sex (male vs. female at birth) and HIV parameters (CDC-C, current viraemia viral load ≥200 and CD4^+^ : CD8^+^ ratio <1) or INSTI and/or TAF-containing regimens on metabolic outcomes, adjusting for age group at study entry (Supplementary Table 1 and Figure 1).

**Table 2:** Estimated effect of INSTI-based antiretroviral thearpy regimens, tenofovir alafenamide-containing regimens and HIV clinical markers on metabolic outcomes, obtained from Bayesian generalized linear regression models (Gaussian link for continuous outcomes; logit link for binary outcomes).

Outcome type	Outcome	Risk factor	Estimate [95% credible interval]^a^	Posterior probability of positive association	Significant
Continuous outcomes	BMI change	INSTI	0.16 [−1,1. 37]	0.60	No
		TAF	−0.72 [−1.94, 0.5]	0.12	No
		INSTI+TAF	−0.07 [−1.7, 1.58]	0.46	No
		Prior CDC-C	0.17 [−1.04, 1.35]	0.61	No
		^**^VL ≥200	0.21 [−1.3, 1.7]	0.60	No
		CD4^+^ : CD8^+^ ratio <1	−0.47 [−1.71, 0.74]	0.22	No
	Fibroscan CAP	INSTI	−2.73 [−26.74, 21.02]	0.41	No
		TAF	−3.79 [−28.1, 19.73]	0.38	No
		INSTI+TAF	−17.46 [−47.65, 13.35]	0.13	No
		Prior CDC-C	−4.19 [−28.62, 19.19]	0.36	No
		VL ≥200	−0.95 [−30.35,29.45]	0.46	No
		CD4^+^ : CD8^+^ ratio <1	1.06 [−23.05, 24.88]	0.54	No
	HbA1c	INSTI	1.73 [−0.28, 3.76]	0.96	No
		TAF	−0.36 [−2.32, 1.65]	0.35	No
		INSTI+TAF	0.19 [−2.51, 2.77]	0.56	No
		Prior CDC-C	−0.34 [−2.28, 1.57]	0.36	No
		VL ≥200	1.4 [-1,3.83]	0.87	No
		CD4^+^ : CD8^+^ ratio <1	0.97 [−0.88, 2.89]	0.84	No
	HOMA-IR	TAF	0.3 [−0.63, 1.2]	0.75	No
		INSTI	0.41 [−0.45, 1.26]	0.82	No
		INSTI+TAF	0.47 [−0.71, 1.62]	0.80	No
		Prior CDC-C	0.29 [−0.54, 1.11]	0.75	No
		VL ≥200	−0.14 [−1.21, 0.88]	0.40	No
		CD4^+^ : CD8^+^ ratio <1	0.19 [−0.72, 1.13]	0.65	No
Binary outcomes	Hepatic steatosis	INSTI	0.58 [0.13, 1.62]	0.12	No
		TAF	1.52 [0.37, 4.07]	0.66	No
		INSTI+TAF	0.42 [0.02, 1.68]	0.09	No
		Prior CDC-C	0.63 [0.17, 1.64]	0.14	No
		VL ≥200	3.39 [0.7, 9.73]	0.92	No
		CD4^+^ : CD8^+^ ratio <1	1.07 [0.28, 2.92]	0.42	No
	MetS	INSTI	3.97 [0.43, 16.26]	0.85	No
		TAF	2.81 [0.29, 12.23]	0.71	No
		INSTI+TAF	7.62 [0.83, 30.85]	0.96	No
		Prior CDC-C	15.02 [0.74, 82.02]	0.95	No
		VL ≥200	1.11 [0.03, 5.09]	0.35	No
		CD4^+^ : CD8^+^ ratio <1	3.38 [0.4, 13.93]	0.79	No
	2 criteria of MetS	INSTI	1.14 [0.25, 3.23]	0.44	No
		TAF	1.35 [0.27, 4.06]	0.53	No
		INSTI+TAF	1.22 [0.11, 4.23]	0.44	No
		Prior CDC-C	3.86 [0.71, 13.9]	0.93	No
		VL ≥200	2.07 [0.29, 6.75]	0.71	No
		CDC 4:8 ratio <1	3.91 [0.75, 13.6]	0.93	No

Each row corresponds to a distinct model, adjusting for age and sex, and models for change in BMI also adjusted for baseline BMI.

a Estimated coefficients and 95% credible intervals on odds ratio scale for binary outcomes.

ART, antiretroviral therapy; CAP, controlled attenuation parameter; CDC-C, Centre for Disease Control category C AIDS diagnostic condition; HbA1c, glycosylated haemoglobin; HOMA-IR, homeostatic model for assessment of insulin resistance; INSTI, integrase strand transfer inhibitor; MetS, metabolic syndrome; TAF, tenofovir alafenamide; **VL, most recent viral load.

## Discussion

In this cohort of youth with PHIV (mean age 21.7 years), rates of MetS were low (7%) although half were overweight or obese, with 48% having dyslipidaemia. Rates of MetS were comparable to PHIV cohorts in France and Thailand but lower than an older cohort in the Unites States (13% MetS; median age 30 years), with MetS prevalence increasing with age in the general population [6,8,9]. Rates of overweight/obesity in 16–24-year-olds in England are estimated at 37%, associated with female sex, black ethnicity and deprivation; risk factors common in this study population [36]. The median weight gain over 26 months was 3 kg, with BMI category overweight/obese moving from 37 to 50%. Obesity rates increase with age; however, recent general population data in England suggest those currently aged 18–24 years have the highest risk of BMI gain compared to older age groups [37]. Increasing youth weight gain is associated with environmental factors (calorie-rich processed food, increasingly sedentary lifestyle) and genetic hereditability results in both metabolic and psychological consequences [38].

While weight gain has been reported in association with INSTIs and TAF, we found no association with adverse metabolic outcomes, including BMI change, suggesting that addressing traditional risk factors is more important for metabolic health than switching ART. Current viraemia and past CDC-C diagnosis were not associated with adverse metabolic outcomes; however, maintaining viral suppression remains a priority for youth where rates of viral suppression are lower than in adults, with uncontrolled viraemia associated with multimorbidity and potential onward transmission [5]. Rates of MetS defined dyslipidaemia approached 50% in this cohort. The Reprieve study led to the recommendation of empirical statin use from 40 years in adults with HHIV [27]. However, there are no recommendations for statins in PHIV despite high rates of dyslipidaemia reported in both high-income and low-income settings (34–70%) and in the context of lifelong inflammation and increased cardiovascular risk [7,10,19,22–25,39]. With the metabolic impact of lifestyle interventions for youth limited and pharmacotherapy currently targeted to older adults with established morbidity, the optimal timing and modality of intervention strategies to prevent cardiovascular disease remains a research priority for this emerging adult population with PHIV [5,10,27,39].

The limitations of this study include the relatively short duration of follow-up, potential selection bias of those consenting to the sub study and variable rates of viral suppression during the study period potentially influencing the impact of ART, although reflecting real-world adherence patterns in this population and the challenges of maintaining viral suppression during this period of life [5]. Major limitations include the low participant number resulting in likely underpowering to study ART interactions and lack of age and ethnically matched HIV-negative youth. However, the lack of effect on outcomes by ART regimen (INSTI vs. other, TAF vs. non-TAF/TDF and INSTI with TAF vs. other) adds to the evidence base underlining the importance of addressing traditional risk factors for adverse metabolic health from early childhood and through into adult life, while maintaining sustained viral suppression.

## Acknowledgements

The writing team would like to thank all the participants who took part in the BONDY-Met Study and the members of the BONDY study group Sara Ayres, Alice Walley, Rebecca Hall, Natalie Kirkhope, Fiona Brown, and Wilbert Ayap.

Authors’ contributions: C.F. conceived the study, recruited participants, collated the data, and wrote the draft article. A.B. performed the statistical analyses and reviewed the article. H.L. recruited participants and reviewed the draft article. S.F. and M.H. recruited patients, reviewed the data and draft article. and are supported by Imperial College NIHR BRC.

Funding: this study was funded by an Investigator led grant from Gilead Sciences (C.F.).

### Conflicts of interest

There are no conflicts of interest.

## Supplementary Material

Supplemental Digital Content

## References

[1] Trachunthong D Tipayamongkholgul M Chumseng S Darasawang W Bundhamcharoen K. Burden of metabolic syndrome in the global adult HIV-infected population: a systematic review and meta-analysis. *BMC Public Health* 2024; 24:2657. 39342258 10.1186/s12889-024-20118-3 PMC11438355

[2] Foster C Ayers S McDonald S Frize G Chhabra S Pasvol S Fidler S. Clinical outcomes post transition to adult services in young adults with perinatally acquired HIV infection: mortality, retention in care and viral suppression. *AIDS* 2020; 34:261–266. 31651427 10.1097/QAD.0000000000002410

[3] Majonga ED Henderson M Ferrand RA. Cardiovascular health in people with perinatally acquired HIV - where do we stand? *Curr Opin HIV AIDS* 2024; 19:348–354. 38935060 10.1097/COH.0000000000000872

[4] López E Sainz T Dirajlal-Fargo S Jao J Pinto J Buchanan AM. Cardiometabolic health burden in pediatric HIV: unmet need in the contemporary antiretroviral therapy era. *Cureus* 2025; 17:e85329. 40621302 10.7759/cureus.85329 PMC12227333

[5] Henderson M Fidler S Foster C. Adults with perinatally acquired HIV; emerging clinical outcomes and data gaps. *Trop Med Inf Dis* 2024; 9:74. 10.3390/tropicalmed9040074 PMC11053933 38668535

[6] Haw NJL Lesko CR Ng DK Lam J Lang R Kitahata MM North American AIDS Cohort Collaboration on Research and Design (NA-ACCORD). Incidence of non-AIDS defining comorbidities among young adults with perinatally acquired HIV in North America. *AIDS* 2024; 38:1366–1374. 38507583 10.1097/QAD.0000000000003892 PMC11211058

[7] Sahagun SJ Yeramosu T Purdy JB Reynolds JC Hadigan CM. Associations between central obesity and lifelong antiviral therapy in adults living with HIV acquired from early childhood. *J Acquir Immune Defic Syndr* 2022; 89:208–214. 34693931 10.1097/QAI.0000000000002841 PMC8752474

[8] Arrive E Viard J-P Salanave B Dollfus C Matheron S Reliquet V ANRS CO19 COVERTE and ENNS study groups. Metabolic risk factors in young adults infected with HIV since childhood compared with the general population. *PLoS One* 2018; 13:e0206745. 30408056 10.1371/journal.pone.0206745 PMC6226109

[9] Aurpibul L Namwongprom S Sudjaritruk T Ounjaijean S. Metabolic syndrome, biochemical markers, and body composition in youth living with perinatal HIV infection on antiretroviral treatment. *PLoS One* 2020; 15:e0230707. 32226033 10.1371/journal.pone.0230707 PMC7105120

[10] Dirajlal-Fargo S Nyathi M Sun S Bonner LB Kahts M Asafu-Agyei NA. Metabolic syndrome and Pathobiological Determination of Atherosclerosis in Youth risk score in adolescents with and without perinatally acquired HIV in the Cape Town Adolescent and Antiretroviral Cohort (CTAAC)-Heart study. *HIV Med* 2025; 26:1239–1250. 40400088 10.1111/hiv.70049 PMC12315067

[11] Gawrieh S Vilar-Gomez E Woreta TA Lake JE Wilson LA Price JC. Prevalence of steatotic liver disease, MASLD, MetALD and significant fibrosis in people with HIV in the United States. *Aliment Pharmacol Ther* 2024; 59:666–679. 38158589 10.1111/apt.17849 PMC10922859

[12] Lemoine M Assoumou L De Wit S Girard PM Valantin MA Katlama C ANRS-ECHAM Group. Diagnostic accuracy of noninvasive markers of steatosis, NASH, and liver fibrosis in HIV-monoinfected individuals at risk of nonalcoholic fatty liver disease (NAFLD): results from the ECHAM Study. *J Acquir Immune Defic Syndr* 2019; 80:e86–e94. 30570529 10.1097/QAI.0000000000001936

[13] Chen BR Pan CQ. Non-invasive assessment of fibrosis and steatosis in pediatric non-alcoholic fatty liver disease. *Clin Res Hepatol Gastroenterol* 2022; 46:101755. 34311134 10.1016/j.clinre.2021.101755

[14] Rose PC Davies C Cotton MF Otwombe K Browne SH Vaida F. Longitudinal controlled attenuation parameter and liver stiffness in children with and without perinatal HIV infection in South Africa. *AIDS* 2024; 38:1638–1647. 38905492 10.1097/QAD.0000000000003964 PMC11317452

[15] Carrasco I Olveira A Lancharro Escosa L Mellado MJ Busca C. Prevalence of nonalcoholic fatty liver disease using noninvasive techniques among children, adolescents, and youths living with HIV. *AIDS* 2022; 36:805–814. 35013082 10.1097/QAD.0000000000003170

[16] Hatleberg CI Ryom L Sabin C. Cardiovascular risks associated with protease inhibitors for the treatment of HIV. *Expert Opin Drug Saf* 2021; 20:1351–1366. 34047238 10.1080/14740338.2021.1935863

[17] Capeau J Lagathu C Béréziat V. Recent data on the role of antiretroviral therapy in weight gain and obesity in persons living with HIV. *Curr Opin HIV AIDS* 2024; 19:14–20. 38078606 10.1097/COH.0000000000000833

[18] Savinelli S Newman E Mallon PWG. Metabolic complications associated with use of integrase strand transfer inhibitors (INSTI) for the treatment of HIV-1 infection: focus on weight changes, lipids, glucose and bone metabolism. *Curr HIV/AIDS Rep* 2024; 21:293–308. 39207722 10.1007/s11904-024-00708-x PMC11486773

[19] Santiprabhob J Tanchaweng S Maturapat S Maleesatharn A Lermankul W Sricharoenchai S. Metabolic disorders in HIV-infected adolescents receiving protease inhibitors. *Biomed Res Int* 2017; 2017:7481597. 28293638 10.1155/2017/7481597 PMC5331476

[20] Glenn J Bennett A Mackie NE Lyall EGH Taylor GPT Fidler S Foster C. The cumulative prevalence of HIV-1 drug resistance in perinatal HIV. *AIDS* 2025; 39:1161–1177. 40235031 10.1097/QAD.0000000000004202 PMC12237116

[21] Eni-Olutu A Mackie N Glenn J Bailey A Bamford A Kenny J. Emerging integrase resistance in an international perinatal virtual clinic. *AIDS* 2025; 39:276–280. 39469738 10.1097/QAD.0000000000004048

[22] Belfrage E Soeria-Atmadja S Navér L. Growth, weight gain and BMI in virally suppressed children on antiretroviral therapy with specific reference to dolutegravir. *BMC Pediatr* 2023; 23:339. 37403042 10.1186/s12887-023-04143-6 PMC10318707

[23] Turkova A White E Mujuru HA Kekitiinwa AR Kityo CM Violari A Dolutegravir as first- or second-line treatment for HIV-1 infection in children. ODYSSEY Trial Team. *N Engl J Med* 2021; 38527:2531–2543. 10.1056/NEJMoa2108793 PMC7614690 34965338

[24] Yeoh DK Campbell AJ Bowen AC. Increase in body mass index in children with HIV, switched to tenofovir alafenamide fumarate or dolutegravir containing antiretroviral regimens. *Pediatr Infect Dis J* 2021; 40:e215–e216. 33847305 10.1097/INF.0000000000003076

[25] Thivalapill N Simelane T Mthethwa N Dlamini S Lukhele B Okello V. Transition to dolutegravir is associated with an increase in the rate of body mass index change in a cohort of virally suppressed adolescents. *Clin Infect Dis* 2021; 73:e580–e586. 33119739 10.1093/cid/ciaa1652 PMC8326552

[26] Rourke J Townsend CL Milanzi E Collins IJ Castro H Judd A. Effectiveness and safety of tenofovir alafenamide in children and adolescents living with HIV: a systematic review. *J Int AIDS Soc* 2023; 26:e26037. 36823283 10.1002/jia2.26037 PMC9950035

[27] Grinspoon SK Fitch KV Zanni MV Fichtenbaum CJ Umbleja T Aberg JA REPRIEVE Investigators. Pitavastatin to prevent cardiovascular disease in HIV infection. *N Engl J Med* 2023; 389:687–699. 37486775 10.1056/NEJMoa2304146 PMC10564556

[28] Triant VA Lyass A Hurley LB Borowsky LH Ehrbar RQ He W. Cardiovascular risk estimation is suboptimal in people with HIV. *J Am Heart Assoc* 2024; 13:e029228. 38761071 10.1161/JAHA.123.029228 PMC11179796

[29] Rajashekhar M Joshi M Mundra A Kolhe R Kirubakaran R Raut AV Nazli Khatib M. Community-based health promotion interventions to reduce risk factors in non-communicable diseases among adolescents and young adults in low- and middle-income countries: a systemic review and meta-analysis. *Public Health* 2025; 243:105714. 40328577 10.1016/j.puhe.2025.03.026

[30] Henderson M Blenkinsop A Ratmann O Cheung M Lyall H Fidler S Foster C. BONDY study group. Bone health in a U.K. cohort of youth living with perinatally acquired HIV-1: a longitudinal study. *J Int AIDS Soc* 2025; 28:e70029. 40904314 10.1002/jia2.70029 PMC12409145

[31] NHS commissioning policy: Tenofovir alafenamide for treatment of HIV-1 in adults and adolescents. Available at: https://www.england.nhs.uk/wp-content/uploads/2017/03/f03-taf-policy.pdf. (Accessed 8 November 2025)

[32] Matthews DR Hosker JP Rudenski AS Naylor BA Treacher DF Turner RC. Homeostasis model assessment: insulin resistance and β-cell function from fasting plasma glucose and insulin concentrations in man. *Diabetologia* 1985; 28:412–419. 3899825 10.1007/BF00280883

[33] Mikolasevic I Orlic L Franjic N Hauser G Stimac D Milic S. Transient elastography (FibroScan®) with controlled attenuation parameter in the assessment of liver steatosis and fibrosis in patients with nonalcoholic fatty liver disease - where do we stand? *World J Gastroenterol* 2016; 22:7236–7251. 27621571 10.3748/wjg.v22.i32.7236 PMC4997649

[34] Anagnostis P. 2025 The metabolic syndrome. BMJ Best practice updated 12 December. Available at: https://bestpractice.bmj.com/topics/en-gb/212/pdf/212/Metabolic%20syndrome.pdf. (Accessed 9 January 2026)

[35] Messina C Albano D Gitto S Tofanelli L Bazzocchi A Ulivieri FM. Body composition with dual energy X-ray absorptiometry: from basics to new tools. *Quant Imaging Med Surg* 2020; 10:1687–1698. 32742961 10.21037/qims.2020.03.02 PMC7378094

[36] NHS England. The Health Survey for England 2022 Part 2. Available at: https://digital.nhs.uk/data-and-information/publications/statistical/health-survey-for-england/2022-part-2/adult-overweight-and-obesity#:~:text=Overweight%20and%20obesity%2C%20by%20age,women%20aged%2055%20to%2074. (Accessed 6 December 25)

[37] Katsoulis M Lai AG Diaz-Ordaz K Gomes M Pasea L Banerjee A. Identifying adults at high-risk for change in weight and BMI in England: a longitudinal, large-scale, population-based cohort study using electronic health records. *Lancet Diabetes Endocrinol* 2021; 9:681–694. 34481555 10.1016/S2213-8587(21)00207-2 PMC8440227

[38] Heymsfield S Wadden TA. Mechanisms, pathophysiology and management of obesity. *N Engl J Med* 2017; 376:254–266. 28099824 10.1056/NEJMra1514009

[39] Henderson M Klastrup V Ahmad S Glenn J Ayres S Jadayel H. Evaluation of cardiovascular risk factors among adults with perinatally acquired HIV. *Open Forum Infect Dis* 2025; 12:ofaf629. 41216424 10.1093/ofid/ofaf629 PMC12596388

