# Supplementary material for: Risk factors associated with adverse metabolic health in youth with perinatally acquired HIV living in the United Kingdom
Source: AIDS. 2026 Jun 2;40(10):1459–66. doi: 10.1097/QAD.0000000000004533 (PMC13566416; doi:10.1097/QAD.0000000000004533)
Supplement: Supplementary file 1 [file aids-40-1459-s001.docx]

**Supplementary Material**

**Supplementary Tables**

| **Outcome type** | **Outcome** | **Predictors** | **Interaction terms** | **Risk factor** | **Estimate [95% credible interval]** | **Posterior probability of positive association** | **Significant** |
| --- | --- | --- | --- | --- | --- | --- | --- |
| **Continuous outcomes** | Change in BMI | INSTI, sex, age group | INSTI * sex | INSTI | 1.04 [-0.77,2.85] | 0.87 | No |
|  |  |  |  | Effect modification of INSTI among girls | -1.5 [-3.88,0.82] | 0.1 | No |
|  |  | TAF, sex, age group | TAF * sex | TAF | -1.44 [-3.27,0.41] | 0.06 | No |
|  |  |  |  | Effect modification of TAF among girls | 1.2 [-1.15,3.55] | 0.85 | No |
|  |  | Prior CDC-C, sex, age group | Prior CDC-C * sex | Prior CDC-C | -0.06 [-1.95,1.81] | 0.47 | No |
|  |  |  |  | Effect modification of Prior CDC-C among girls | 0.4 [-2.02,2.81] | 0.62 | No |
|  |  | Viremia (VL>=200), sex, age group | Viremia (VL>=200) * sex | Viremia (VL>=200) | 0.25 [-2.23,2.68] | 0.58 | No |
|  |  |  |  | Effect modification of Viremia (VL>=200) among girls | -0.08 [-3.23,3] | 0.48 | No |
|  |  | CDC4:8 ratio<1, sex, age group | CDC4:8 ratio<1 * sex | CDC4:8 ratio<1 | -1.07 [-2.95,0.78] | 0.13 | No |
|  |  |  |  | Effect modification of CDC4:8 ratio<1 among girls | 1.04 [-1.35,3.63] | 0.79 | No |
|  | Fibroscan CAP score | INSTI, sex, age group | INSTI * sex | INSTI | 14.27 [-24.42,51.32] | 0.78 | No |
|  |  |  |  | Effect modification of INSTI among girls | -28.53 [-76.82,18.38] | 0.11 | No |
|  |  | TAF, sex, age group | TAF * sex | TAF | 0.46 [-36.29,37.15] | 0.5 | No |
|  |  |  |  | Effect modification of TAF among girls | -7.2 [-54.63,40.16] | 0.39 | No |
|  |  | Prior CDC-C, sex, age group | Prior CDC-C * sex | Prior CDC-C | -10.14 [-47.04,25.26] | 0.28 | No |
|  |  |  |  | Effect modification of Prior CDC-C among girls | 10.85 [-36.29,57.4] | 0.68 | No |
|  |  | Viremia (VL>=200), sex, age group | Viremia (VL>=200) * sex | Viremia (VL>=200) | -8.91 [-55.61,40.64] | 0.36 | No |
|  |  |  |  | Effect modification of Viremia (VL>=200) among girls | 12.9 [-49.23,72.57] | 0.66 | No |
|  |  | CDC4:8 ratio<1, sex, age group | CDC4:8 ratio<1 * sex | CDC4:8 ratio<1 | 6.01 [-32.01,43.54] | 0.63 | No |
|  |  |  |  | Effect modification of CDC4:8 ratio<1 among girls | -7.41 [-55.56,42.71] | 0.37 | No |
|  | HbA1c | INSTI, sex, age group | INSTI * sex | INSTI | 1.93 [-1.18,4.89] | 0.9 | No |
|  |  |  |  | Effect modification of INSTI among girls | -0.35 [-4.25,3.67] | 0.44 | No |
|  |  | TAF, sex, age group | TAF * sex | TAF | -0.58 [-3.61,2.62] | 0.36 | No |
|  |  |  |  | Effect modification of TAF among girls | 0.35 [-3.75,4.53] | 0.57 | No |
|  |  | Prior CDC-C, sex, age group | Prior CDC-C * sex | Prior CDC-C | -0.82 [-3.85,2.06] | 0.3 | No |
|  |  |  |  | Effect modification of Prior CDC-C among girls | 0.8 [-2.95,4.78] | 0.65 | No |
|  |  | Viremia (VL>=200), sex, age group | Viremia (VL>=200) * sex | Viremia (VL>=200) | 4.37 [0.38,8.31] | 0.98 | No |
|  |  |  |  | Effect modification of Viremia (VL>=200) among girls | -4.89 [-9.89,0.04] | 0.03 | No |
|  |  | CDC4:8 ratio<1, sex, age group | CDC4:8 ratio<1 * sex | CDC4:8 ratio<1 | 1.91 [-1.17,5.08] | 0.89 | No |
|  |  |  |  | Effect modification of CDC4:8 ratio<1 among girls | -1.59 [-5.62,2.52] | 0.21 | No |
|  | HOMA-IR | INSTI, sex, age group | INSTI * sex | INSTI | 0.43 [-0.95,1.77] | 0.73 | No |
|  |  |  |  | Effect modification of INSTI among girls | -0.03 [-1.8,1.73] | 0.48 | No |
|  |  | TAF, sex, age group | TAF * sex | TAF | 0.42 [-0.94,1.82] | 0.74 | No |
|  |  |  |  | Effect modification of TAF among girls | -0.25 [-1.99,1.51] | 0.39 | No |
|  |  | Prior CDC-C, sex, age group | Prior CDC-C * sex | Prior CDC-C | -0.3 [-1.62,1.04] | 0.32 | No |
|  |  |  |  | Effect modification of Prior CDC-C among girls | 1.03 [-0.72,2.69] | 0.88 | No |
|  |  | Viremia (VL>=200), sex, age group | Viremia (VL>=200) * sex | Viremia (VL>=200) | 0.43 [-1.4,2.16] | 0.68 | No |
|  |  |  |  | Effect modification of Viremia (VL>=200) among girls | -0.94 [-3.2,1.35] | 0.21 | No |
|  |  | CDC4:8 ratio<1, sex, age group | CDC4:8 ratio<1 * sex | CDC4:8 ratio<1 | 0.02 [-1.32,1.36] | 0.51 | No |
|  |  |  |  | Effect modification of CDC4:8 ratio<1 among girls | 0.27 [-1.48,2.06] | 0.62 | No |
| **Binary outcomes** | Hepatic steatosis | INSTI, sex, age group | INSTI * sex | INSTI | 1.48 [0.17,5.65] | 0.5 | No |
|  |  |  |  | Effect modification of INSTI among girls | 0.46 [0.02,2.36] | 0.11 | No |
|  |  | TAF, sex, age group | TAF * sex | TAF | 3.56 [0.45,14.91] | 0.84 | No |
|  |  |  |  | Effect modification of TAF among girls | 0.64 [0.03,3.27] | 0.17 | No |
|  |  | Prior CDC-C, sex, age group | Prior CDC-C * sex | Prior CDC-C | 0.44 [0.04,1.68] | 0.08 | No |
|  |  |  |  | Effect modification of Prior CDC-C among girls | 6.41 [0.25,33.41] | 0.81 | No |
|  |  | Viremia (VL>=200), sex, age group | Viremia (VL>=200) * sex | Viremia (VL>=200) | 4.35 [0.3,18.09] | 0.83 | No |
|  |  |  |  | Effect modification of Viremia (VL>=200) among girls | 2.56 [0.08,14.07] | 0.5 | No |
|  |  | CDC4:8 ratio<1, sex, age group | CDC4:8 ratio<1 * sex | CDC4:8 ratio<1 | 0.64 [0.08,2.43] | 0.18 | No |
|  |  |  |  | Effect modification of CDC4:8 ratio<1 among girls | 7.86 [0.4,39.86] | 0.87 | No |
|  | Metabolic syndrome | INSTI, sex, age group | INSTI * sex | INSTI | 1.66 [0,11.7] | 0.25 | No |
|  |  |  |  | Effect modification of INSTI among girls | 1417.88 [0.32,7873.14] | 0.92 | No |
|  |  | TAF, sex, age group | TAF * sex | TAF | 1.01 [0,7.45] | 0.19 | No |
|  |  |  |  | Effect modification of TAF among girls | 923.51 [0.32,3815.79] | 0.91 | No |
|  |  | Prior CDC-C, sex, age group | Prior CDC-C * sex | Prior CDC-C | 1.75 [0,12.34] | 0.24 | No |
|  |  |  |  | Effect modification of Prior CDC-C among girls | 10829.8 [1.26,41871.12] | 0.98 | No |
|  |  | Viremia (VL>=200), sex, age group | Viremia (VL>=200) * sex | Viremia (VL>=200) | 204.09 [0.26,805.46] | 0.91 | No |
|  |  |  |  | Effect modification of Viremia (VL>=200) among girls | 0.07 [0,0.39] | 0.01 | No |
|  |  | CDC4:8 ratio<1, sex, age group | CDC4:8 ratio<1 * sex | CDC4:8 ratio<1 | 1.09 [0,7.64] | 0.18 | No |
|  |  |  |  | Effect modification of CDC4:8 ratio<1 among girls | 1546.79 [0.37,5376.2] | 0.94 | No |
|  | 2 criteria of metabolic syndrome | INSTI, sex, age group | INSTI * sex | INSTI | 1.62 [0.04,8.56] | 0.39 | No |
|  |  |  |  | Effect modification of INSTI among girls | 5.93 [0.08,32.35] | 0.6 | No |
|  |  | TAF, sex, age group | TAF * sex | TAF | 1.27 [0.05,6.1] | 0.35 | No |
|  |  |  |  | Effect modification of TAF among girls | 6.58 [0.13,37.58] | 0.7 | No |
|  |  | Prior CDC-C, sex, age group | Prior CDC-C * sex | Prior CDC-C | 5.28 [0.2,30.52] | 0.74 | No |
|  |  |  |  | Effect modification of Prior CDC-C among girls | 5.76 [0.08,32.24] | 0.64 | No |
|  |  | Viremia (VL>=200), sex, age group | Viremia (VL>=200) * sex | Viremia (VL>=200) | 5.27 [0.09,30.18] | 0.71 | No |
|  |  |  |  | Effect modification of Viremia (VL>=200) among girls | 3.48 [0.02,18] | 0.37 | No |
|  |  | CDC4:8 ratio<1, sex, age group | CDC4:8 ratio<1 * sex | CDC4:8 ratio<1 | 5.42 [0.19,30.31] | 0.69 | No |
|  |  |  |  | Effect modification of CDC4:8 ratio<1 among girls | 5.98 [0.08,39.16] | 0.68 | No |

*Supplementary Table 1: Estimated effect of INSTI-based ART regimens, TAF-containing regimens and HIV clinical markers on metabolic outcomes, for interactions between treatment and sex. Linear regression (for continuous outcomes) and logistic regression (for binary outcomes).
* estimated coefficients and 95% credible intervals on odds ratio scale for binary outcomes.*

**Supplementary Figure 1**

**Figure 1: Estimated effect of INSTI-based ART regimens, TAF-containing regimens and HIV clinical markers on metabolic outcomes, obtained from Bayesian generalised linear regression models (Gaussian link for continuous outcomes; logit link for binary outcomes). Each row corresponds to a distinct model, adjusting for age and sex, and models for change in BMI also adjusted for baseline BMI.**

* Estimated coefficients and 95% credible intervals on odds ratio scale for binary outcomes*.*

ART; antiretroviral therapy, INSTI; integrase strand transfer inhibitor, TAF; tenofovir alafenamide, BMI; body mass index, HbA1c; glycosylated haemoglobin, CAP; controlled attenuation parameter. HOMA-IR; homeostatic model for assessment of insulin resistance, MetS; metabolic syndrome.
